# *CASZ1*: Current Implications in Cardiovascular Diseases and Cancers

**DOI:** 10.3390/biomedicines11072079

**Published:** 2023-07-24

**Authors:** Heng Jian, Ansgar Poetsch

**Affiliations:** 1 Queen Mary School, Nanchang University, Nanchang 330006, China; 2 School of Basic Medical Sciences, Nanchang University, Nanchang 330006, China

**Keywords:** *CASZ1*, cell differentiation, cancer, cardiovascular disorders

## Abstract

Castor zinc finger 1 (CASZ1) is a C2H2 zinc finger family protein that has two splicing variants, CASZ1a and CASZ1b. It is involved in multiple physiological processes, such as tissue differentiation and aldosterone antagonism. Genetic and epigenetic alternations of *CASZ1* have been characterized in multiple cardiovascular disorders, such as congenital heart diseases, chronic venous diseases, and hypertension. However, little is known about how CASZ1 mechanically participates in the pathogenesis of these diseases. Over the past decades, at first glance, paradoxical influences on cell behaviors and progressions of different cancer types have been discovered for CASZ1, which may be explained by a “double-agent” role for CASZ1. In this review, we discuss the physiological function of *CASZ1*, and focus on the association of *CASZ1* aberrations with the pathogenesis of cardiovascular diseases and cancers.

## 1. Introduction

Castor zinc finger 1 (CASZ1), also known as zinc finger protein 693 (ZNF693), is a transcription factor that is evolutionarily conserved and participates in multiple embryonic development and physiological processes. In 1992, Mellerick and Cui first discovered the drosophila homolog *Castor* (i.e., *ming*) and observed impaired axon alignments and reduced axon density in *Castor* mutant Drosophila embryos, proposing a role in central nervous system (CNS) development and cell specification [1,2]. Later, researchers found that the mice homolog *CST* is widely expressed in the CNS and developing heart [3], and it was not until 2006 that the human *Castor* gene (*CASZ1*) was first discovered, which encodes two isoforms namely CASZ1a (i.e., hcasz11) and CASZ1b (i.e., hcasz5) [4].

CASZ1 belongs to the C2H2-type zinc finger protein family characterized by the Cys-Cys-His-His (C2H2) zinc finger motif, which is homologous to the Xenopus transcription factor IIIA (TFIIIA) [4]. In this large protein family, the zinc finger domains are responsible for binding DNA on the major grooves to exert regulatory effects in gene transcription and, therefore, diverse physiological processes such as development and cellular behaviors. There is no exception for CASZ1; for instance, in 2008, Christine et.al first found that the Xenopus homolog of CASZ1 is critical for cardiomyocyte differentiation and heart morphogenesis, and Charpentier et al. discovered its role in vessel patterning and endothelial cell behaviors [5,6]. CASZ1 has also been found to regulate aldosterone synthesis and repress the activation of aldosterone target genes, suggesting that it may participate in blood pressure regulation [7,8,9].

Studies have also characterized the involvement of *CASZ1* in disease conditions, mostly cardiovascular disorders and cancers. Clinical cases displayed *CASZ1* mutations in congenital heart diseases, while GWAS (Genome-wide association studies) and EWAS (Epigenome-Wide Association Studies) discovered the association of *CASZ1* single nucleotide polymorphisms (SNPs) or aberrant epigenetic status with chronic venous disease (CVD), hypertensive diseases, and all-cause mortality for cardiovascular disease populations [10,11,12,13]. Interestingly, in cancers, CASZ1 was found to function as pro- and anti-oncogenic, which depends on cell/tumor types: neuroblastoma (NB) is the earliest and mostly used tumor model for CASZ1 studies, in which CASZ1 shows suppressive effects on NB cell proliferation and metastasis, and promotes cell differentiation [14]; however, in epithelial ovarian cancer, glioma and lung adenocarcinoma, CASZ1 is oncogenic and related to a worse prognosis [15,16,17]. *CASZ1* downstream genes in different cancers include differentiation markers, cell cycle proteins, adhesion molecules and cytoskeleton molecules. For some downstream targets, CASZ1 can regulate on their gene loci the histone modifications, chromatin accessibility, as well as the gain and loss of super enhancers, which are highly potent transcriptional activators [18,19,20].

Although multiple functions and disease relevance have been revealed for *CASZ1*, there is still a large gap in knowledge about the underlying molecular mechanisms in physiological and pathological processes. In addition, whether different diseases possibly share similar pathogenic mechanisms caused by *CASZ1* anomalies is unknown due to a lack of evidence. In this review, we discuss the physiological function of this gene, and its involvement in cardiovascular diseases and cancers. We also analyzed and compared CASZ1 functions as a tumor suppressor or activator. Though roles of *CASZ1* in vascular endothelial cells has been reviewed [21], this is the first comprehensive review of *CASZ1* with a broader presentation of the pathophysiological implications.

## 2. CASZ1 Molecular Structure and Physiological Functions

### 2.1. Molecular Structure

**Figure 1:**
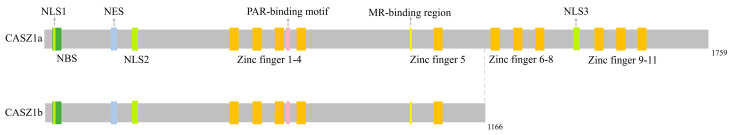
Schematic of CASZ1a, CASZ1b protein sequences highlighting domain structure and important motifs. The two isoforms share the NLS1 (2329 AA), NES (176–192 AA), NLS2 (232–248 AA), NuRD complex binding site (NBS, 21–45 AA), PAR-binding motif (640–650 AA), MR-binding region (968–972 AA) and ZF1–5 (490–515 AA, 551–575 AA, 610–634 AA, 668–692 AA, 1031–1055 AA, respectively). CASZ1a additionally has six zinc fingers ZF6–11 (1182–1206 AA, 1242–1266 AA, 1300–1324 AA, 1457–1481 AA, 1515–1537 AA, 1571–1595 AA, respectively) and a NLS3 (1401–1418 AA). The localization of all zinc fingers was acquired by analyzing the CASZ1a sequence using SMART tool [29]. (http://smart.embl-heidelberg.de/) (accessed on 5 July 2023).

The *CASZ1* gene is located at chromosome 1p 36.22 and is transcribed into *CASZ1a* and *CASZ1b* pre-mRNAs from the same promoter [4]. The pre-mRNA contains 16 and 21 exons, respectively, with their first three spliced into the 5′UTR and the remaining constituting open-reading frame and 3′UTR of mature RNA [4,22]. The protein product CASZ1a is 1759 amino acids in length, and is identical to CASZ1b in the first 1166 amino acids [4] (Figure 1). In their shared region (namely CASZ1b), lie five characteristic TFIIIA class C2H2 zinc fingers (ZFs), among which the highly conserved ZF1–4 are essential for tertiary structure maintenance and DNA binding, carrying out the transcriptional functions of CASZ1, while ZF5 is seldom required for transcriptional activities [22,23]. However, there are two nuclear localization signals (NLS1, NLS2) and a nuclear export signal (NES), which co-determine the nuclear location of CASZ1 proteins [22,24]. Some specific regions in CASZ1b are necessary for protein–protein interactions: amino acid (AA) sequence 21–45 binds to metastasis-associated 1 family member 2 (MTA2) and histone deacetylase 1 (HDAC1), which are subunits of the nucleosome-remodeling and deacetylase (NuRD) complex that mediates histone deacetylation and chromosome remodeling [23]. The poly-ADP ribose (PAR) binding motif (AA 640–650) interacts with histone H3 and DNA repair proteins, such as poly (ADP-Ribose) polymerase 1 (PARP1), X-ray repair cross complementing 5 (XRCC5), and the replication protein A1 (RPA1) [23]. The NURD and PAR binding regions are essential for the transcriptional regulation effect of CASZ1, since deletion variants within these regions showed impaired promotion of transcription for target genes [23]. In addition, the PAR binding motif is partly responsible for CASZ1 recruitment to DNA breaks by interacting with the single-stranded break repair protein PARP1 [25]. Furthermore, there is an LXXLL region (amino acids: LGSLL) that binds to the C-terminus of mineralocorticoid receptors (MRs) [8]. Compared with CASZ1b, CASZ1a has an additional NLS region (NLS3) and six zinc fingers (ZF6–11), which probably allow more potent transcriptional effects on target genes [4,26]. According to Uniprot BLAST (https://www.uniprot.org/blast) (accessed on 2 June 2023) and BLAT programs (http://www.genome.ucsc.edu/) (accessed on 2 June 2023), CASZ1b is conserved widely in chordates and *Drosophila melanogaster*, with the highest homology in zinc finger sequences (ZF1–4) [22,27,28]. CASZ1a homologs are less conserved but also exist in mammals, birds, and amphibians [22,28].

### 2.2. CASZ1 Expression Patterns in Embryonic and Adult Tissues

*CASZ1* is ubiquitously expressed in various embryonic tissues. During mouse heart development, CASZ1 is first detected in the primary heart field, and is continually present in the endocardium and myocardium of the whole heart, but not in endothelial cells and epicardium [30]. It also presents in somite and limb bud, which further gives rise to bones, muscles, and subcutaneous tissues of trunks and limbs, respectively [30,31]. During neurogenesis, *CASZ1* expression initiates in neural crests and neural tubes, and is maintained in dorsal root ganglia (DRG), spinal cord, retina, brain, and cranial ganglia, particularly at both afferent neurons (DRG sensory neuron, rod photoreceptor) and interneurons (spinal excitatory interneurons, amacrine cells) [3,32,33,34]. Moreover, naïve CD4^+^ T cells also express *CASZ1* because it is required for their differentiation toward Th17 cells [35]. These studies strongly indicate that CASZ1 is involved in diverse development processes. In human adult tissues, *CASZ1* is expressed in the heart, lung, testis, colon, stomach, small intestine, liver, pancreas, kidney, skeletal muscle, and adrenal cortex [4,9,36]. The existence of tissue-specific methylated regions (tDMRs) on the *CASZ1* promoter enables differential expression levels among adult tissues [36]. *CASZ1* mRNA was not detected in adult human brains and lowly presented in adult mice brains [4,26], speculatively due to the decline of *CASZ1*-expressing stem cells as brain matures.

Subcellularly, CASZ1 is usually localized in the cell nucleus, consistent with its role in transcription regulation. In cardiomyocytes, vestibular and spiral ganglion neurons, CASZ1 colocalizes with promyelocytic leukemia (PML) bodies, a superstructure composed of protein complexes in the nuclear matrix that participates in DNA repair, transcriptional regulation, stemness maintenance, and apoptosis [30,34,37]. The colocalization possibly suggests an interaction with PML bodies and involvement of CASZ1 in these processes, but further studies are required to confirm this hypothesis.

### 2.3. Heart Morphogenesis

To study its roles in heart development, *CASZ1*-depleted mice were generated either through Cre-loxP system or by inserting a truncating reporter sequence into the *CASZ1* gene [30,31]. Although *CASZ1* haploinsufficiency mice did not display obvious growth defects, a knockout of both alleles caused hypoplastic myocardium and ventricular defects with subsequent edema and mortality after E14.5, suggesting that *CASZ1* is critical for heart morphogenesis [30,31]. CASZ1 has been confirmed to interact with T-box transcription factor 20 (TBX20), and double haploinsufficiency of both genes induced dilated cardiomyopathy and cardiac fibrosis in mice [38]. Additionally, Xenopus with CASZ1 deletion on congenital heart disease 5 protein-interacting region (CHD5-interacting region, 785–998 AA) failed to mediate proper heart field fusion, heart tube looping, and chamber formation [39]. These results collectively suggests that CASZ1 cooperates with other transcription factors to regulate gene expression for heart morphogenesis in vertebrates.

**Figure 2:**
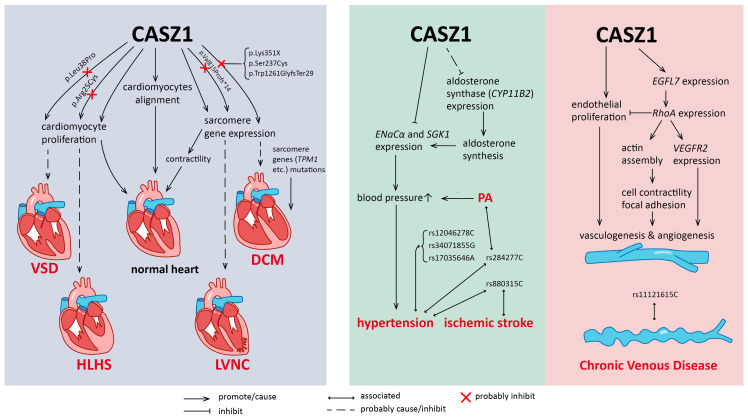
CASZ1 physiological function in cardiovascular development and association of *CASZ1* mutations or single nucleotide polymorphisms (SNPs) with cardiovascular diseases. Left panel: role of CASZ1 in heart development and *CASZ1* mutations in congenital heart disease pathogenesis. Mutations are shown as amino acid alterations (p.Arg25Cys, etc.). TPM1: tropomyosin 1; VSD: ventricular septal defect; HLHS: hypoplastic left heart syndrome; LVNC: left ventricular noncompaction; DCM: dilated cardiomyopathy. Right panel: role of CASZ1 in aldosterone antagonism and vessel patterning, as well as *CASZ1* SNPs associated with hypertensive diseases and chronic venous disease. *CASZ1* SNPs are shown as reference SNPs with the last letter representing nucleotides (rs284277C, etc.). CYP11B2: cytochrome P450 family 11 subfamily B member 2; ENaCα: epithelial Na(+) channel subunit alpha; SGK1: serum/glucocorticoid regulated kinase 1; EGFL7: EGF-like domain multiple 7; RhoA: Ras homolog family member A; VEGFR2: vascular endothelial growth factor receptor 2.

CASZ1 plays a crucial role in cardiomyocyte growth and function (Figure 2, left panel). During chamber formation stage (E12.5), CASZ1 promotes cardiomyocyte proliferation, while *CASZ1* knockout mice had declined cardiomyocyte number, reduced G1-S transition rates, and increased cells expressing phosphorylated retinoblastoma-associated protein (pRB) [30]. In Xenopus, CASZ1 is necessary to initiate the differentiation of ventral midline precursor cells that give rise to cardiomyocytes in the outer ventricular curvature, but inhibit their proliferation; additionally, *CASZ1* is scarcely expressed in most dividing cardiomyocytes during chamber formation [5,40]. These results collectively suggest different functions of CASZ1 between mammalian and amphibian heart development. The second role of CASZ1 lies in the maintenance of cardiomyocyte arrangement, since *CASZ1* depletion in Xenopus caused a decline of tight junction protein 1 (TJP1/ZO-1) and claudin-5, accompanied by consequentially widened cellular gaps and ruptures of basement membranes [39]. Moreover, CASZ1 is required for the expression of myofibril genes and the proper arrangements of α-actinin and F-actin, which are the critical components of sarcomeres [31].

Microarray results from *CASZ1* knockout mice confirmed a global alteration in gene transcription [31]. Consistent with the proliferative effect, cell cycle regulators such as tumor protein P63 (*TP63*) and transforming growth factor beta 3 (*TGFB3*) displayed increased mRNA levels. There was also an elevation of extracellular matrix genes and integrin subunit gene transcripts, namely integrin subunit alpha 7 (*ITGA7*) and integrin subunit alpha 10 (*ITGA10*), but decreased creatine kinase M-type (*CKM*) and sarcomere markers like actin alpha 1 (*ACTA1*), troponin I2 (*TNNI2*). However, subunits of L-type calcium channel, voltage-gated potassium channels and Na^+^/K^+^-ATPase were aberrantly expressed after CASZ1 depletion [31]. Therefore, CASZ1 may serve as a necessary determinant of cardiomyocyte growth, organization, contractility, and electrical conduction throughout heart morphogenesis.

### 2.4. Skeletal Muscle Differentiation

Early studies on Xenopus found *CASZ1* expressed in somite and terminally differentiated skeletal muscles [5,40], suggesting that *CASZ1* has a possible function in skeletal muscle production. Recently, this hypothesis has been substantiated in mice, since in differentiating myoblasts *CASZ1* is upregulated and activates *ACTA1*, *CKM* expression, promoting differentiation and fusion into myotubes [18]. The study also proposed that CASZ1 forms a feed-forward autoregulatory circuit with transcription factors myogenic differentiation 1 (MYOD) and myogenin (MYOG), each of them boosting transcriptions of themselves and the other two by binding to super enhancer regions on the gene loci [18]. These three genes can directly promote the transcription of differentiated skeletal muscle marker genes, such as tropomyosin 1 (*TPM1*), troponin T2 (*TNNT2*), troponin C2 (*TNNC2*) and troponin I1 (*TNNI1*) for sarcomere function [18]. Therefore, their expression is required for the skeletal muscle differentiation. In embryonal rhabdomyosarcoma, this regulatory circuit is inhibited by the aberrantly activated MAPK (mitogen-activated protein kinase) pathway, thus arresting the tumor cells at an undifferentiated status [18]. This will be further discussed in the following sections.

### 2.5. Neuronal Differentiation

In embryonic mice adrenal medullary, CASZ1 levels increase with sympathoblasts differentiating into chromaffin cells [19]. Genes co-expressed with *CASZ1* include those for synapse functions such as synaptophysin (*SYP*) and chromogranin A (*CHGA*) [19]. Similarly in neuroblastoma cell lines, the *CASZ1* mRNA level increases concomitantly when neuroblastoma cells are induced to differentiate [4]. Restoration of *CASZ1* level not only induces morphological differentiation of NB cells toward neurons, but also upregulates neuronal markers, such as nerve growth factor receptor (*NGFR*) and tyrosine hydroxylase (*TH*), while downregulating mesenchymal gene expressions [19]. Given that neuroblastoma is derived from neural crest cells or sympathoadrenal precursors, these results collectively suggest that CASZ1 probably promotes cell differentiations in sympathoadrenal lineage. Additionally, *CASZ1* is also found to be expressed in both neural progenitors and differentiated neurons in the dorsal spinal cord and dorsal root ganglia [33]. It has been proven that CASZ1 controls the temporal differentiation pattern of neural progenitors, in particular for retina progenitor differentiation toward rod photoreceptor and bipolar cells [32]. Taken together, CASZ1 participates in the differentiation of diverse neural types, but the molecular mechanisms await elucidation.

### 2.6. Vessel Patterning

Blood vessels, another mesodermal organ, is also a target organ of CASZ1 throughout morphogenesis. Vessel patterning initiates when mesodermal cells of blood islands migrate and differentiate to form the primary vascular plexus, which then undergoes tubulogenesis and sprouts into subordinate vessels [41]. *CASZ1* knockdown in the Xenopus embryo caused a reduced primary vascular plexus, impaired posterior cardinal vein tubulogenesis, and delayed intersomitic vessel extension, suggesting that CASZ1 has a crucial role throughout vessel patterning processes [6]. Further studies on human umbilical vein endothelial cells (HUVEC) found that CASZ1 promotes endothelial behaviors that facilitate vessel patterning, including cell proliferation, a cobblestone-like cell shape, contractility maintenance, and adhesion to extracellular substrates [6].

Mechanistically, the influence of CASZ1 on endothelial behavior can be explained by its direct upregulation of EGF-like domain multiple 7 (*EGFL7*), which in turn activates transcription of Ras homolog family member A (*RHOA*) [6] (Figure 2, right panel). As a GTPase, RhoA plays a key role in the assembly of actin stress fibers and focal adhesions, which promote cell contractility and adhesion on the extracellular matrix [42]. Additionally, RhoA contributes to expression of vascular endothelial growth factor receptor 2 (*VEGFR2*), the initiator of angiogenesis pathways [43]. However, RhoA can also be suppressive for vessel patterning, as it was recently found to inhibit tubulogenesis and HUVEC proliferation, yet induce endothelial cell death [44]. Therefore, further investigations are needed to confirm whether CASZ1 acts solely through this RhoA pathway or is involved in other angiogenic pathways to promote vessel patterning.

### 2.7. Aldosterone Regulation

Blood pressure can be elevated by aldosterone, a hormone that binds to mineralocorticoid receptors (MRs) intracellularly and activates Na^+^ channel expression in renal tubule epithelial cells, thus promoting Na^+^ and water reabsorption, and blood volume increase. Zona glomerulosa cells, and aldosterone-producing cell clusters in normal adrenal cortex or those affected by aldosterone producing adenoma, can express aldosterone synthase (i.e., CYP11B2), which is responsible for the final step of aldosterone synthesis [45].

Interestingly, recent studies suggest that in the nuclei of renal tube epithelial cells, CASZ1 interacts with MR through the LGSLL sequence [8]. Such interaction indirectly suppresses transcription activations of MR target genes, including serum/glucocorticoid regulated kinase 1 (*SGK1*) and its downstream effector epithelial Na(+) channel subunit alpha (*ENaCα*), which is critical for Na^+^ reabsorption [8]. *CASZ1* mRNA is also detected in zona glomerulosa cells and aldosterone-producing cell clusters [9]. Overexpression of *CASZ1* reduces aldosterone synthase (*CYP11B2*) and aldosterone levels in adrenocortical carcinoma cells (H295R-S2), suggesting that it probably inhibits *CYP11B2* expression and the consequential aldosterone synthesis [9]. These two studies hint at a potential role of CASZ1 in blood pressure regulation by antagonizing both function and synthesis of aldosterone (Figure 2, right panel).

### 2.8. DNA Damage Repair

CASZ1 has a PAR-binding motif that interacts with DNA repair proteins such as PARP1 and XRCC5 [23]. PARP1, which mediates repair of single-strand breaks, is partially responsible for recruiting CASZ1 to a DNA damage region [25]. Although CASZ1 is not required for the initiation of the DNA repair process, it is necessary for cell survival under radiation-induced DNA damage, which might be attributed to the activation of other protective genes in damage responses [25]. This suggests that CASZ1 may prevent the initiation of tumorigenesis induced by unrepaired DNA damage.

## 3. CASZ1 in Cardiovascular Diseases

### 3.1. Heart Abnormalities

Since CASZ1 plays a crucial role in heart morphogenesis, it is reasonable that loss-of-function mutations could lead to cardiomyopathy or congenital heart diseases (CHD) with structural defects, as were observed in *CASZ1*-depleted mice or Xenopus (Figure 2 left panel, Table 1). Dilated cardiomyopathy (DCM) is characterized by an expanded left ventricle with a thinned ventricular wall and impaired contractility, and is often associated with mutations in sarcomere genes such as *TPM1*, *TNNT2* and *MYH7* (Myosin Heavy Chain 7) [46]. A patient with dilated cardiomyopathy was found carrying a point mutation (p.Lys351X), which lies in a conserved region of CASZ1, causing truncation from the 350th amino acid that impairs its transcription-activating ability [47]. However, a de novo mutation (mutation with the first appearance in a family) that induces CASZ1a truncation has been observed in another DCM case [48]. Since CASZ1 regulates sarcomere alignments and transcription of sarcomere genes during heart morphogenesis, it is possible that these genes are dysregulated in these *CASZ1*-mutated patients and consequently lead to sarcomere dysfunction and DCM.

Left ventricular noncompaction (LVNC) can also result from sarcomere gene mutations and features excessive myocardial trabeculae and deepened intertrabecular recesses [49]. One LVNC patient has a mutation that introduces a frameshift and truncates both CASZ1a and CASZ1b, affecting the 5th to 16th zinc finger and the CHD5-interacting region. This likely impairs its transcriptional activity and binding to CHD5 protein, and therefore disrupts the normal process of heart morphogenesis [50].

**Table 1:** *CASZ1* mutations and the association with congenital heart diseases.

Disorders	Genetic Aberration	Mutation Description
ventricular septal defect	Heterozygous missense mutation c.113T>C (p.Leu38Pro)	Dominant inheritance with complete penetrance. Luciferase-reporter assay using tyrosine hydroxylase (TH) promotor showed a reduced transcriptional promotive activity [11].
dilated cardiomyopathy	1. Heterozygous nonsense mutation c.1051A>T (p.Lys351X)	Dominant inheritance with complete penetrance. Luciferase-reporter assay using TH promotor showed a reduced transcriptional promotive activity [47].
	2. Homozygous missense mutation c.710C>G (p.Ser237Cys)	Patient also has left ventricular noncompaction. Heterozygote parents do not have CHD phenotypes [51].
	3. Heterozygous frameshift mutation: c.3781del (p.Trp1261GlyfsTer29)	De novo mutations that cause CASZ1a truncation from the 1280th amino acid and subsequent loss ofZF6-ZF11 and nuclear localization signal 3 (NLS3), without affecting CASZ1b structures [48].
left ventricular noncompaction	Heterozygous frameshift mutation c.2443_2459delGTGGGCACCCCCAGCCT (p.Val815Profs*14)	De novo mutations that cause CASZ1a CASZ1b truncation from the 827th amino acid. The ZF5 and congenital heart disease 5 protein (CHD5) binding region of CASZ1b is lost [50].
hypoplastic left heart syndrome	Heterozygous missense mutation c.73C>T (p.Arg25Cys)	Heterozygote mother has a bicuspid aortic valve phenotype [52]. The mutation affects NLS1 and hinders CASZ1 entrance to nucleus. The same mutation is also found in ERMS cases [18].

Moreover, a missense mutation in a non-essential region that impairs CASZ1 transcriptional activity has been found in patients with ventricular septal defect (VSD), similar to one phenotype of *CASZ1*-depleted mice [11]. A hypoplastic left heart syndrome (HLHS) patient (together with his bicuspid aortic valve mother) carried mutations affecting NLS1 regions, thus disabling CASZ1 from entering cell nuclei [52]. Mechanisms for both VSD and HLHS are not completely understood but given that cardiomyocytes are essential building blocks of the ventricular septum and wall, we propose a possible pathogenesis where these two mutants failed to promote normal cardiomyocyte proliferation, resulting in the underdevelopment of cardiac structures (Figure 2, left panel).

Most patients in the aforementioned cases were diagnosed in early ages, indicating that the defects induced by *CASZ1* are congenital, but not secondary to other pathological conditions. Although *CASZ1* mutants are usually heterozygotes, one missense mutation (within NLS2-coding sequence) is recessively inherited and displays DCM and LVNC only when it is homozygous [51]. This suggests that *CASZ1* haploinsufficiency is not always sufficient to predispose CHDs, consistent with the fact that *CASZ1* haploinsufficiency mice had no cardiac phenotypical change [30]. Of note, CASZ1 locus lies in 1p36, suggesting it is also a candidate gene involved in 1p36 deletion syndrome (deletion of 1p36 region on one chromosome), which is characterized by congenital heart abnormalities with multiorgan defects such as intellectual impairment and vision defects [53]. However, more studies are needed to confirm whether the loss of *CASZ1* contributes to the cardiac defects in 1p36 deletion syndrome.

### 3.2. Hypertension and Venous Diseases

Multiple GWAS (Genome-wide association studies) studies involving Europeans or Asians have discovered various *CASZ1* risk alleles, which probably cause *CASZ1* misexpression linked to hypertension, although the mechanisms are still unclear [10,54,55,56,57]. These risk alleles include rs880315C (C-allele of SNP rs880315), rs12046278C, rs34071855G, and rs17035646A (Figure 2, right panel). Particularly, SNP rs284277C is a risk allele for primary aldosteronism (PA), a hypertensive disease mostly caused by aldosterone producing adenoma, where *CASZ1* aberrant upregulation was observed [9]. Another allele, rs880315C, is related to higher systolic and diastolic pressures in patients of different body mass indexes (BMI) [54]. However, by studying gene methylation levels in patients with ischemic stroke, researchers later found that rs880315C correlates to hypomethylation at one CpG site (cg12760995) which, together with another 10 hypomethylated CpG sites, is associated with higher blood pressure or risks for ischemic stroke (IS) [58]. Both rs284277C and rs880315C suggest a phenomenon where *CASZ1* is upregulated or hypomethylated in primary aldosteronism or hypertension, which seems contradictory to the aforementioned hypothesis that CASZ1 regulates blood pressure by antagonizing aldosterone synthesis and functioning. We propose that these two SNPs might be scarcely potent on transcription repression of aldosterone targets, or there are other feedback pathways promoting *CASZ1* expression due to pre-existing hypertensive conditions.

Of note, hypertension can be secondary in pathophysiology to neuroblastoma because some neuroblastomas have noradrenergic features and synthesize excessive catecholamines that cause blood pressure elevation. However, CASZ1 is suppressive to neuroblastoma, and is frequently downregulated in neuroblastoma cell lines and clinical samples [14,26]. This proposes another pathogenic mechanism: *CASZ1* downregulation predisposes neuroblastoma, which in turn leads to hypertension. Therefore, there might not exist a simple connection between CASZ1 levels and hypertension.

### 3.3. Chronic Venous Diseases

Chronic venous diseases (CVD) describe a pathological state that originates from venous blood pooling, which causes endothelial activation, inflammation response, and venous wall remodeling [59]. Intrinsic structural defects of the vein wall are also a predisposing factor for vein disorders. Clinically, CVD is staged according to the Clinical, Etiologic, Anatomic, and Pathophysiologic (CEAP) standard, which contains C0 (No visible or palpable signs of venous disease), C1 (Telangiectasia or reticular veins), C2 (Varicose veins), C3 (Edema), C4 (skin alternations), C5 (Healed venous ulcer) and C6 (Active venous ulcer) [59]. *CASZ1* SNP rs11121615 C-allele (rs11121615C) is a risk allele for chronic venous disease (CVD), especially for those with CEAP stage larger than C2 (Figure 2, right panel) [12,60,61,62]. However, whether this SNP is a cause for CVD remains unknown. ChIP-seq demonstrated that the region near rs11121615C has H3K27ac, H3K4me1 modifications, and elevated enhancer activity, which may lead to *CASZ1* upregulation [12]. Paradoxically, another study using polymerase chain reaction (PCR) confirmed the significant downregulation of *CASZ1* in venous leg ulcer samples compared with normal controls [63]. Since CASZ1 determines the physiological patterning of veins, it is possible that both excessive and insufficient expression of *CASZ1* disturbs the physiological regulation of vessel development and impairs venous wall structures, potentially increasing the risk for CVD.

## 4. *CASZ1* in Cancers

**Figure 3:**
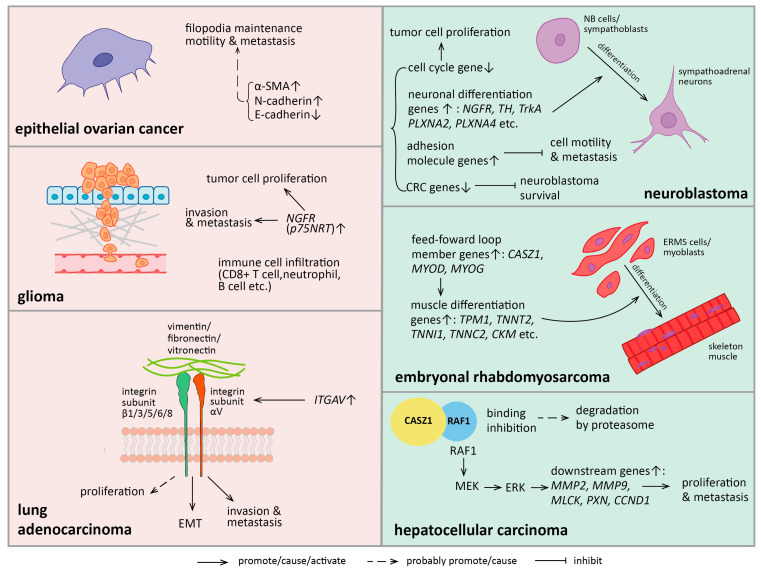
“Double-agent” role of *CASZ1* in cancer progression. The figure shows molecular and cellular changes when *CASZ1* is expressed in different tumor cells. The left half shows its oncogenic role in lung adenocarcinoma, glioma, and epithelial ovarian cancer. The right half shows its tumor suppressive role in neuroblastoma embryonal rhabdomyosarcoma and hepatocellular carcinoma. *CASZ1* impacts the proliferation, epithelial-to-mesenchymal transition (EMT) and metastasis of cancer cells through interacting with regulators or influencing gene expressions. α-SMA: actin alpha 2, smooth muscle; NGFR: nerve growth factor receptor; ITGAV: integrin subunit αV; TH: tyrosine hydroxylase; TrkA: tropomyosin-related kinase A; PLXNA2: plexin A2; PLXNA4: plexin A4; CRC: core regulatory circuitry; MYOD: myogenic differentiation 1; MYOG: myogenin; TPM1; tropomyosin 1; TNNT2: troponin T2; TNNI1: troponin I1; TNNC2: troponin C2; CKM: creatine kinase M-type; RAF1: Raf-1 proto-oncogene; MEK: MAP kinase kinase; ERK: MAP kinase; MMP2: metalloproteinase 2; MMP9: metalloproteinase 9; MLCK: myosin light chain kinase; PXN: paxillin; CCND1: cyclin D1. ↑: increased gene expression or protein level; ↓: decreased gene expression or protein level.

Studies have revealed the association of CASZ1 with multiple cancer types. However, CASZ1 can act either as a tumor repressor or an oncogene, depending on the cellular context (Figure 3, Table 2). Mechanistically, the impact of CASZ1 on cancer cell behaviors is attributed to its regulation of genes commanding proliferation, differentiation, or adhesion. Moreover, mutations and aberrant expression patterns of CASZ1 might be causative of cancer development, suggesting the potential of a future application as a prognostic indicator.

### 4.1. Neuroblastoma

The tumor suppressive effect of CASZ1 was initially characterized in neuroblastoma (NB) patients, where a *CASZ1* expression anomaly was observed: NB patients are usually accompanied by DNA hypermethylation, histone deacetylation, or 1p loss of heterozygosity that reduces the CASZ1 level, or cytoplasmic mislocalization of the CASZ1 protein that disables its transcriptional role [14,24,26,64]. Both CASZ1 insufficiency and cytoplasmic localization are associated with poor prognostic markers, such as “unfavorable” Shimada histology and MYCN proto-oncogene (*MYCN*) amplification [14,24].

In the NB cell line, SY5Y, an overexpression of *CASZ1* not only caused morphological changes, including formation of extensions, growth cones, and dense aggregates in the cytoplasm, but also reduced tumor cell proliferation and motility in vitro [14]. Moreover, the implantation of *CASZ1*-overexpressing NB cell lines in vivo ultimately generated tumors with impaired growth [14]. Both CASZ1a and CASZ1b have the independent ability to inhibit tumor cell proliferation in vitro and tumor growth in mice, but CASZ1a induces a higher level of neuronal biomarker and is more extensively expressed in NB cell lines than CASZ1b, suggesting it is the mainstay of tumor suppression [26].

Mechanistically, CASZ1 directly promotes transcription of neural differentiation markers *NGFR*, *TH* and upregulates cell adhesion genes protocadherin 1 (*PCDH1*), CD9 molecule (*CD9*) and intercellular adhesion molecule 2 (*ICAM2*), which have suppressive effects on NB cell migration [14,74,75]. Moreover, it activates the metastasis suppressor clusterin (*CLU*), while repressing the transcription of the MYC proto-oncogene (*MYC*) [14,19]. G1-to-S transition proteins like cyclin E and cyclin-dependent kinase 2 (CDK2), or G2-to-M transition proteins like cyclin B1 are widely suppressed by CASZ1, thereby explaining its inhibitory effect on NB cell proliferations [14,19,76]. The core regulatory circuitry (CRC) transcription factors of NB, including paired like homeobox 2B (PHOX2B), GATA binding protein 3 (GATA3), heart and neural crest derivatives expressed 2 (HAND2), ISL LIM homeobox 1 (ISL1), achaete-scute family bHLH transcription factor 1 (ASCL1) and T-box transcription factor 2 (TBX2), form an autoregulated feed-forward loop that strengthens their own expression, but switches on genes of the oncogenic pathway and is crucial for maintaining sympathoadrenal cell states and cell survival [19,77,78,79]. CASZ1 suppresses CRC transcription factor expression by reducing H3K27ac signals and enhancer activities on their gene loci, thereby inhibiting neuroblastoma oncogenesis [19]. Taken together, CASZ1 acts as a multidirectional suppressor in neuroblastoma.

The downregulation of *CASZ1* in neuroblastoma is not only attributed to the genetic anomaly (1p36 LOH), but also epigenetic modifications. The enhancer of zeste homolog 2 (EZH2), the enzymatic subunit of polycomb complex 2, directly binds to the transcription start site of *CASZ1* loci and mediates the repressive histone modification H3K27me3, thereby downregulating *CASZ1* expression [80]. Treatment of neuroblastoma cell lines with EZH2 antagonists, such as valproic acids or DZNep, can restore *CASZ1* expression, inhibiting tumor cell proliferation and promoting morphological differentiation [80,81]. Histone modification is another mechanism for *CASZ1* transcription inhibition, and one possible mediator is the CRC transcription factor HAND2, which directly binds on *CASZ1* introns and promoter [14,19,64]. When *HAND2* was knocked down in sympathoadrenal NB cell lines, *CASZ1’s* mRNA level increased, accompanied by an elevated H3K27ac marker but a declined H3K27me3 marker around the transcription start site and HAND2-binding introns [19]. T-box transcription factor 2 (TBX2) also directly binds to CASZ1 loci and induces CASZ1 upregulation when knocked down, suggesting that it is another contributor to *CASZ1* suppression [19].

### 4.2. Other Cancers

The tumor suppressive effects of CASZ1 are also evident in rhabdomyosarcoma and hepatocellular carcinoma. Rhabdomyosarcoma is the most common malignant soft tissue sarcoma in children and originates from neoplastic striated muscles [82]. In subtype embryonal rhabdomyosarcoma, the aberrantly upregulated MAP kinase kinase (MEK) suppresses *CASZ1* expression and impairs the autoregulated pathway composed of *CASZ1*, *MYOD,* and *MYOG*, thus inhibiting tumor cells from differentiating into skeletal muscles [18]. However, *CASZ1* point mutations, which putatively affect its transcriptional function, are also detected in some patients [18] (Table 2). Restoration of *CASZ1* expression in ERMS increased H3K27ac binding to the super enhancers of myogenic regulators *MYOD*, *MYOG* and *MEF2D* (myocyte enhancer factor 2D), as well as striated muscle differentiation marker *TNNI1* and *TPM1* [18]. This suggests that, in line with normal myoblasts, CASZ1 also induces the differentiation of ERMS toward skeletal muscles. In hepatocellular carcinoma (HCC) samples, *CASZ1* is downregulated compared to normal tissues and mainly localizes in the cytoplasm [65]. Raf-1 proto-oncogene (RAF1), a MAPK pathway component that is aberrantly activated in HCC, can indirectly downregulate cyclin D1 (*CCND1*), matrix metallopeptidase 2 (*MMP2*) and cytoskeleton genes like paxillin (*PXN*), which are crucial for tumor cell migration, focal adhesion and consequential metastasis [65,83] (Figure 3). CASZ1 binding and destabilization of RAF1 probably result in degradation by proteasome, thereby suppressing HCC cell proliferation and metastasis [65]. HCC patients with lower levels of CASZ1 had a poorer prognosis and higher possibility of relapse [65].

In contrast, several studies have described the oncogenic function of CASZ1 supporting tumor proliferation, epithelial-to-mesenchymal transition (EMT), and metastasis, suggesting a “double-agent” role among different cancer types. In epithelial ovarian cancer (EOC) cell lines, *CASZ1* knockdown impaired filopodia formation, whereas overexpression showed an upregulation of mesenchymal gene N-cadherin, α-SMA (actin alpha 2, smooth muscle) and a downregulation of E-cadherin at protein level [16]. This probably causes the increase in cell motility and invasiveness in *CASZ1*-knockdown EOC cells [16]. Consistently, the injection of *CASZ1*-knockdown EOC cells into mice reduced the number of lung metastasis nodules compared with control [16]. CASZ1 also enhances the proliferation and invasion of glioma cells by directly upregulating *NGFR* (i.e., *p75NTR*), which is considered an oncogene for glioma [17]. Additionally, CASZ1 is positively related to immune cell infiltration and expression of proinflammatory interleukins or chemokines, which establish a microenvironment that probably promotes cancerous properties and glioma progression [17]. A recent study found that CASZ1 promoted EMT and metastasis of lung adenocarcinoma (LUAD) by directly binding to two sites on the promoter of the integrin subunit αV (*ITGAV*) gene, thereby upregulating its expression [15]. ITGAV can assemble into dimers with integrin subunit β1/3/5/6/8, interacting with ECM such as fibronectins and activating intracellular pathways for cancer cell invasiveness; it also promotes cell proliferation in breast cancer, although this has not been substantiated in LUAD, but might explain the oncogenic effect of *CASZ1* on LUAD [15,84,85]. However, the association between *CASZ1* expression and LUAD prognosis is still controversial because different results were acquired when bioinformatically analyzing the GEO series and TCGA database [15,66] (Table 2).

Other studies also found *CASZ1* mutations or expression aberrations in different cancers, but the effect on cell behavior and cancer progression remains elusive (Table 2). In urothelial carcinomas, cervical carcinomas, and oral squamous cell carcinomas, various types of *CASZ1* mutations were observed, while in esophageal cancer and prostate cancer, *CASZ1* was found to be hypermethylated or silenced by miRNA, respectively, and therefore decreased in expression [71,72,73]. Low-level *CASZ1* expression is associated with a worse prognosis in colorectal cancer and renal clear cell carcinoma [68,69]. These findings suggest that *CASZ1* might have a global participation in different tumor types.

### 4.3. CASZ1 Cellular Contexts-Dependent Functions and “Double-Agent” Roles

“Double agents” refers to genes that have both tumor suppressive and oncogenic effects [86]. Since CASZ1 has been shown to be tumor suppressive in several tumor types but oncogenic in others, we propose it has “double-agent” role, which is ultimately decided by cellular contexts (Figure 3). For example, CASZ1 suppresses NB partially due to upregulation of *NGFR,* which encodes a Fas/TNF-R family receptor that mediates apoptosis of neuroblastoma cells in the presence of nerve growth factor (NGF) [87]. In glioma, although *NGFR* is also upregulated, the empty binding status ultimately induces remodeling of actin cytoskeleton that potentiates glioma cells for invasiveness [88]. It is possible that differences of ligand binding, NGFR protein modifications, distributions, or concentrations of the interacting protein between these two cell types result in these different outcomes. In carcinoma cells, CASZ1 upregulates the genes for cell migration or invasiveness, represented by *ITGAV*, which encodes integrin subunit αV for fibronectin or vitronectin interactions, and *α-SMA*-encoding actin stress fiber that is essential for EOC cell motility [15,16]. However, in ERMS with mesenchymal properties, CASZ1 binds and directly downregulates genes for muscle cell movement, as shown by ingenuity pathway analysis [18]. CASZ1 also downregulates invasiveness-related genes like integrin subunit alpha 7 (*ITGA7*), integrin subunit alpha 10 (*ITGA10*), aggrecan (*ACAN*) and collagen type II alpha 1 chain (*COL2A1*) in cardiomyocytes [31], which have similar cell properties and expression profiles as ERMS cells. Therefore, it is likely that CASZ1 enhances the migration property of carcinomas, while it inhibits that in sarcomas.

Sometimes CASZ1 can adopt different mechanisms but cause similar outcomes in cancer progression. In NB cells, CASZ1 activates neuronal differentiation genes but represses genes for striated muscle development [19]. In ERMS cells, however, CASZ1 upregulates muscle genes, whereas it downregulates neurogenic genes like SRY-box transcription factor 4 (*SOX4*) and *NGF* [18]. Both functions promote tumor cell differentiation, but toward different tissue types in the aforementioned cases. Specifically, the myogenic regulators (*MYOD, MYOG*) that cooperate with CASZ1 to upregulate muscle genes are expressed in *CASZ1*-restored ERMS but absent in NB cells. Therefore, it is probable that the abundance differences in CASZ1 co-activators induce such opposed outcomes of gene activation.

## 5. Other Pathogenic Roles of CASZ1

According to a recent study, CASZ1 plays an anti-inflammatory role in osteoarthritis by downregulating interleukin 6 (*IL-6*) and tumor necrosis factor-alpha (*TNF-α*), while preventing apoptosis of chondrocytes [89], which are unable to regenerate. However, whether *CASZ1* is differentially expressed in an osteoarthritis environment is still unclear. Additionally, *CASZ1* is upregulated in the intrauterine growth-restricted placenta, but the introns are hypermethylated in large-for-gestational-age infants, suggesting that it probably plays a role in intrauterine fetus growth [90]. Principal component and correlation analysis for Alzheimer’s disease demonstrated that several rare *CASZ1* mutations were associated with altered synaptic function [91]. In idiopathic pulmonary fibrosis (IPF) samples, a general hypermethylation of differentially methylated regions (DMRs) of *CASZ1* was observed, while immunohistochemistry demonstrated an overall downregulation in airway epithelial cells but upregulation in alveolar type II cells [92]. Further studies characterized 21 differentially expressed genes in *CASZ1* siRNA-treated human airway epithelial cell lines, indicating that CASZ1 might be involved in pathological pathways of IPF and affect its progression [92]. These studies suggest that *CASZ1* is also involved in different pathological processes beyond tumors and cardiovascular diseases.

Since CASZ1 functions in Th17 cell production, and immune modulations of osteoarthritis, it is possible to propose a regulatory effect on both innate and adaptive immunity during other pathogenic processes. In atherosclerosis and heart failure, activities of different T cell types and cytokines are always observed [93,94], which can affect the severity of cardiovascular injuries. Future studies should explore whether CASZ1 regulates immune cell activities, such as cytokine productions or Th17 cell conversions in cardiovascular diseases.

## 6. Conclusions

*CASZ1* is involved in multiple physiological processes such as cell differentiation, development, and aldosterone antagonism. However, these processes are possibly impaired in various cardiovascular diseases and cancers due to *CASZ1* genetic or expression aberrations. *CASZ1* is differentially expressed in various cancer types and plays “double-agent” roles in their progression. It is also a potential prognostic indicator and possible molecular target in future cancer treatment. However, more studies are necessary to verify the role of CASZ1 in these reported tumor types, and whether *CASZ1* mutations/SNPs are sufficient to cause hypertension and CVD also requires further elucidation. However, little is known about the molecular mechanism of *CASZ1* aberration underlying these cancers and cardiovascular diseases; despite multiple studies characterizing gene expression changes by controlling the CASZ1 level, it is still unclear whether CASZ1 directly regulates the transcription of some differentially expressed genes. Although *CASZ1* c.73C>T is observed in ERMS and hypoplastic left heart syndrome [18,52], it is too early to say that both diseases share a common pathogenesis; considering current understanding, a common pathogenesis would be the exception and not the rule.

One limitation of this review is the experimental access to *CASZ1* expression information from embryonic and adult human tissues owing to ethical reasons. Since it was not the focus of this manuscript, little was mentioned about CASZ1 roles and molecular mechanisms in retinogenesis.

Future studies should further explore the upstream and downstream genes to characterize CASZ1 molecular pathways in different cellular contexts. There is potential to apply *CASZ1* in the prenatal genetic examination of CHDs and pediatric tumors or as a prognostic indicator for cancers. CASZ1 targeting therapies may also be a future opportunity but are challenged by the intracellular localization of CASZ1 protein and the “double-agent” role, which dictates the development of cell context-specific drugs.

## Figures and Tables

**Table 2:** CASZ1 genetic or expression aberrations and their indications in various cancer types.

Tumor Types	Functions	Expression Aberration or Mutations	Prognostic/Diagnostic Indications
neuroblastoma	Tumor Suppressive	CASZ1 insufficiency due to loss of heterozygosity (LOH) caused by 1p36 deletion, hypermethylation and altered histone modifications [14,64]. CASZ1a is higher expressed than CASZ1b. Protein can mislocalize to cytoplasm [24,26].	High level of *CASZ1a* or *CASZ1b* mRNA independently indicates a good prognosis [26]. Most NB cells with cytoplasm localization of CASZ1 has “unfavorable” Shimada histology [24].
embryonal rhabdomyosarcoma	Tumor Suppressive	AA substitution R25C caused by mutation (c.73C>T) which is located in nuclear localization signal 1 (NLS1), preventing CASZ1 from entering the nucleus. Other mutations cause AA substitution G676C (within ZF4 region), E323D, and M1129T [18].	/
hepatocellular carcinoma	Tumor Suppressive	Lower expression level compared to normal liver tissue [65].	Low-level expression associated with poor prognosis [65].
lung adenocarcinoma	Not Sure	Highly methylated and lowly expressed in lung adenocarcinoma tissue [66]. Higher expressed in more metastatic cell lines [15].	TCGA-LUAD analysis: High-level methylation, low-level expression correlates to poor prognosis [66]. GEO series (GSE31210) analysis: low-level expression correlates to good prognosis [15].
esophageal squamous cell carcinoma	Not Sure	Three highly methylated CpG sites found on *CASZ1* loci [67].	Hypermethylation of *CASZ1* cell-free plasma DNA is a diagnostic indicator [67].
colorectal cancer	Not Sure	The expression level is lower than that of surrounding normal tissues [68].	Low-level expression suggests poor prognosis [68].
renal clear cell carcinoma	Not Sure	Expression differences with normal samples are not clear [69].	Low-level expression in nucleus suggests poor prognosis [69].
androgen- independent prostate cancer	Not Sure	miR-151a-5p bind to the 3′UTR region and inhibit transcription. Upregulation of miR-151a-5p levels may decrease CASZ1 expression [70].	/
cervical carcinomas	Not Sure	Human papilloma virus 16 integrates to the intron between exons 20 and 21, upstream *CASZ1a* splicing acceptor, causing *CASZ1a* spliced from virus E1 open reading frame (ORF) to exon 21 [71].	/
oral squamous cell carcinoma	Not Sure	Missense, nonsense, frameshift and splice acceptor mutation were found [72].	/
urothelial carcinoma	Not Sure	Rare variants: *CASZ1* exon 1 and exon 2 fuse with DNA fragmentation factor subunit alpha (*DFFA*) exon 3 [73].	/
epithelial ovarian cancer	Oncogenic	*CASZ*1 is upregulated in different histology classes. CASZ1a is more extensively expressed than CASZ1b [16].	/
glioma	Oncogenic	CASZ1 expression is upregulated with lower methylation level, than normal brain tissue [17].	High-level expression or low-level methylation suggest poor prognosis [17].

## Data Availability

No new data were created.

## Abbreviations

AA	amino acid
CASZ1	castor zinc finger 1
CHD	congenital heart disease
CVD	chronic venous disease
EOC	epithelial ovarian cancer
ERMS	embryonal rhabdomyosarcoma
HCC	hepatocellular carcinoma
ITGAV	integrin subunit alpha V
LUAD	lung adenocarcinoma
MR	mineralocorticoid receptors
MYOD	myogenic differentiation 1
MYOG	myogenin
NB	neuroblastoma
NGFR	nerve growth factor receptor
SNP	single-nucleotide polymorphism
ZF	zinc finger
